# Immunological evaluation of herbal extracts commonly used for treatment of mental diseases during pregnancy

**DOI:** 10.1038/s41598-023-35952-5

**Published:** 2023-06-14

**Authors:** Moritz Winker, Antoine Chauveau, Martin Smieško, Olivier Potterat, Alexander Areesanan, Amy Zimmermann-Klemd, Carsten Gründemann

**Affiliations:** 1 Translational Complementary Medicine, Department of Pharmaceutical Sciences, University of Basel, Basel, Switzerland; 2 Division of Pharmaceutical Biology, Department of Pharmaceutical Sciences, University of Basel, Basel, Switzerland; 3 Computational Pharmacy, Department of Pharmaceutical Sciences, University of Basel, Basel, Switzerland

**Keywords:** Immunology, Applied immunology, Lymphocytes

## Abstract

Nonpsychotic mental diseases (NMDs) affect approximately 15% of pregnant women in the US. Herbal preparations are perceived a safe alternative to placenta-crossing antidepressants or benzodiazepines in the treatment of nonpsychotic mental diseases. But are these drugs really safe for mother and foetus? This question is of great relevance to physicians and patients. Therefore, this study investigates the influence of St. John’s wort, valerian, hops, lavender, and California poppy and their compounds hyperforin and hypericin, protopine, valerenic acid, and valtrate, as well as linalool, on immune modulating effects in vitro. For this purpose a variety of methods was applied to assess the effects on viability and function of human primary lymphocytes. Viability was assessed via spectrometric assessment, flow cytometric detection of cell death markers and comet assay for possible genotoxicity. Functional assessment was conducted via flow cytometric assessment of proliferation, cell cycle and immunophenotyping. For California poppy, lavender, hops, and the compounds protopine and linalool, and valerenic acid, no effect was found on the viability, proliferation, and function of primary human lymphocytes. However, St. John’s wort and valerian inhibited the proliferation of primary human lymphocytes. Hyperforin, hypericin, and valtrate inhibited viability, induced apoptosis, and inhibited cell division. Calculated maximum concentration of compounds in the body fluid, as well as calculated concentrations based on pharmacokinetic data from the literature, were low and supported that the observed effects in vitro would probably have no relevance on patients. In-silico analyses comparing the structure of studied substances with the structure of relevant control substances and known immunosuppressants revealed structural similarities of hyperforin and valerenic acid to the glucocorticoids. Valtrate showed structural similarities to the T cells signaling modulating drugs.

## Introduction

Nonpsychotic mental diseases (NMDs) include depression, anxiety disorders, somatoform, or dissociative disorders, reactions to severe traumatic stresses, and adjustment disorders and affect approximately 15% of pregnant women in the US^1^. A cross-sectional survey from Switzerland showed that more than half (51.3%) of the participants dealt with mental symptoms during pregnancy, namely insomnia (42.9%), anxiety (17.8%), and depressive moods (9.9%)^2^. During pregnancy, NMDs can have devastating consequences for both mother and child, such as spontaneous abortion, preterm delivery, preeclampsia, low birth weight of the child, and a higher risk for developing postnatal depression^3–6^. The treatment of NMDs in pregnancy is challenging, as therapeutics such as antidepressants (including selective serotonin reuptake inhibitors (SSRIs)) and benzodiazepines (γ- aminobutyric acid type A (GABAA) receptors receptor modulators) can cross the placenta^7^ and influence its functionality^8^. The therapy of pregnant women with antidepressants and benzodiazepines thus affects both mothers and unborn children, as depicted by an increased risk of spontaneous abortion or preterm delivery^9–11^. Moreover, antidepressants potentially affect the maternal immune system^12,13^. According to studies, the fear of possible teratogenic effects leads to insufficient treatment of 86% of pregnant women with NMDs^14^.

Pregnant women have started focusing on herbal medicines, as they are perceived as a safe alternative to synthetic medicines with possible adverse effects. A multinational study involving 9,459 women from 23 countries showed that, on average, 28% of pregnant women took herbal medicines^15^. In a further Swiss survey, as many as 89.9% of pregnant women stated that they used herbal preparations^2^. More than half took them to treat mild NMDs (53.6%)^2^.

Pregnancy is immunologically characterized as an exceptional state of avoidance of harmful immune reactions against the allogeneic fetus, well-balanced with the protection of mother and child from pathogens. In short, the following immunological changes—especially changes in T cell activity—occur as a result of pregnancy: increased estradiol and progesterone levels of pregnancy can promote Th2 immune responses, characterized by the Th2 cytokines interleukin-4 (IL-4) and interleukin-5 (IL-5)^16–19^. Progesterone was also shown to increase interleukin-10 (IL-10) secretion^20^. Regulatory T cells (Tregs) are crucial to avoid fetal rejection^21^. There is evidence that the proportion of Tregs in the circulation is increased during pregnancy^22,23^ and that estrogen elevates the expression of the transcription factor forkhead box P3 (FoxP), a marker for Tregs^24^. In addition to changes in the T cell proportion and function, other functions of the immune system are altered to ensure a higher baseline activity of innate lymphoid cells with an elevated potential to promote inflammatory responses on one hand and a stronger potential to downregulate the adaptive immune response on the other hand. The increase in particles in the circulation of pregnant women requires increased antigen uptake by monocytes, stabilized by lower antigen presentation^25,26^. Neutrophils show higher basal activity in vitro. However, the effector functions of neutrophils, monocytes, and B cells are reduced (reviewed in^27^). Finally, an altered glycosylation of the antibodies^28–30^ and an elevated activity of the complement system^31,32^, well-balanced with an increased level of regulatory factors, is described^33–37^. This delicate equilibrium is essential for a successful pregnancy.

The aim of the present study was to evaluate the T cell modulating effects of five herbal preparations, namely St. John’s wort (*Hypericum perforatum* L., Hypericaceae), valerian (*Valeriana officinalis* L., Caprifoliaceae), hops (*Humulus lupulus* L., Cannabaceae), lavender (*Lavandula angustifolia* Mill., Lamiaceae), and California poppy (*Eschscholzia californica* Cham., Papaveraceae), which are popular in the treatment of NMDs—also in pregnant women. None of these preparations is recommended by the EMA due to the lack of conclusive data, especially in pregnancy^38^. Herbal medications containing St. John’s wort are very popular in the treatment of mild to moderate depression. Compared to conventional medications (SSRIs), St. John’s wort preparations had fewer side effects with comparable efficiency^39^. The anti-inflammatory properties of St. John’s wort have also been described. St. John’s wort inhibits the leukocytic enzyme myeloperoxidase, which is responsible for a target-oriented oxidative burst^40,41^. In a pilot study, St. John’s wort was shown to provide relief for patients with mild to moderate psoriasis, indicated by reduced scaling and thickness of erythema^42^. The positive effects of St. John’s wort are possibly based on an inhibition of lipid inflammatory mediators’ production (which increases vascular permeability) and an inhibition of cytokines, which can lead to the reduction of edema and inflammation^43,44^. In addition, it has been shown that hyperforin inhibits the proliferation of epidermal cells and keratinocytes^45,46^. St. John’s wort modulates serotonin reuptake, which is associated with an improvement of depression on one hand and an increased activity of natural killer cells on the other hand^47^. On the other hand, St. John´s wort has been shown to be a strong inducer of CYP-family enzymes which can lead to changes in the pharmacokinetics of co-administered drugs^48^.

Combined preparations of hops and valerian showed an improvement in patients suffering from sleep disorders^49,50^. In general, valerian is one of the most commonly used medications during pregnancy^15^. In addition to the sleep-improving effect, preclinical studies have shown anxiolytic and antidepressant-like effects^51^. Little is known about the influence of valerian on the immune system. Only an increased activity of innate immunity through the activation of the AMP-activated protein kinase (AMPK) signaling pathway and the induction of reactive oxygen species (ROS) have been described^52^.

The influence of hops on the immune system is conceivable because studies have revealed a suppression of the proliferation and development of IL-2 activated killer cells and cytotoxic T lymphocytes by xanthohumol present in hops^53^. Moreover, xanthohumol inhibited the production of Th1 cytokines (IL-2, IFN-γ and TNF-α) by splenic T cells^53^.

Lavender is used in the form of essential oil in the treatment of anxiety and restlessness as well as sleep disorders^54,55^. The effect of lavender therapy on anxiety disorders was comparable to conventional therapy consisting of antidepressants (SSRI paroxetine) and benzodiazepines (low-dose lorazepam)^56,57^. Due to its immunological effects, lavender is also used as a natural remedy for various inflammatory disorders. A recent study showed that lavender and the compound linalool are potent inhibitors of pro-inflammatory cytokine production in human monocytic cells^58^. Lavender further suppressed the production of Th2 cytokines in the mouse model^59^. One study even investigated the impact of lavender (as an aromatherapy massage) on stress levels and immune function in pregnant women. Results showed that lavender therapy resulted in a significantly higher salivary IgA level (also long-term) and a lower cortisol level^60^. California poppy is used for sleep disorders and stress symptoms. The affinity of unknown ingredients to benzodiazepine receptors and an associated anxiolytic effect has been described^61,62^. Otherwise, California poppy has not been researched extensively at the present time, and its immunological effects are unknown.

In the present study, we aimed to evaluate the herbal drugs St. John’s wort, valerian, hops, lavender, and California poppy on toxicity and genome toxicity aspects, as well as the basic functionality of T lymphocytes in vitro. This is highly relevant to avoid the multifactorial and sensitive balance of fetal tolerance and the immunological protection of mother and child.

## Results

### Effects of selected plant extracts and compounds on the viability of primary human lymphocytes

**Figure 1:**
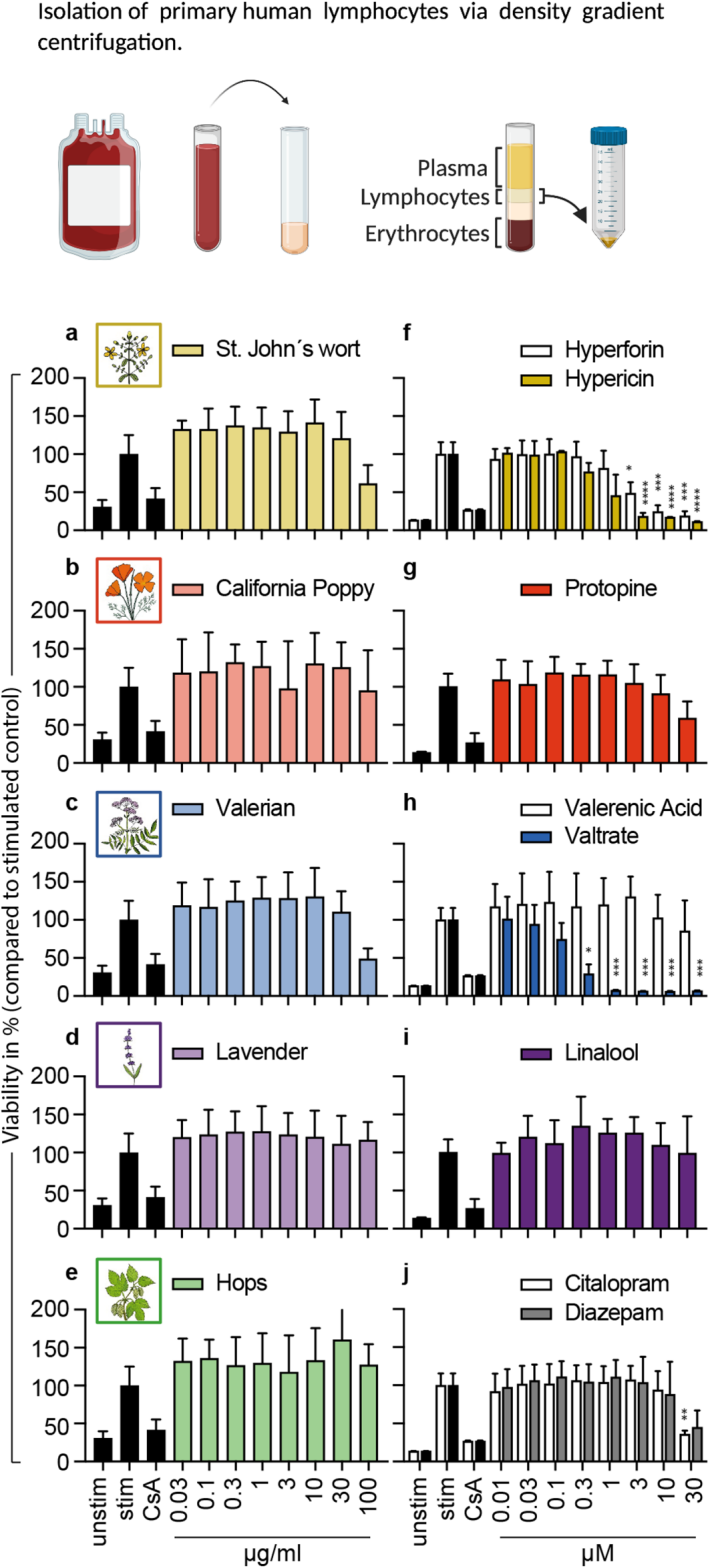
Effects of extracts and compounds on cell viability of primary human lymphocytes. Primary human lymphocytes were stimulated with anti-CD3 and anti-CD28 mAbs and incubated for 72 h with medium (unstim., stim.), cyclosporin A (CsA; 4.16 μM), extracts or compounds. The percentage of viable cells was compared and normalized to the stimulated control and depicted as mean ± standard deviation. *n* = 3; *p < 0.05; **p < 0.01; ***p < 0.005.

A toxic influence on immune system cells would have severe consequences for the immunological balance in pregnancy. Thus, we first determined the influence of the selected substances on the viability of primary human lymphocytes. Because our workflow was based on primary human cells, we isolated primary human lymphocytes from the venous blood of voluntary blood donors and treated them with plant extracts or single compounds in the respective experiments (Fig. 1). We chose a concentration range from 0.03 µg/mL (extracts), or 0.01 µM (compounds) to the control concentration of 100 µg/mL (extracts), or 30 µM (compounds). The synthetic compounds citalopram and diazepam were used as controls. Both controls showed a concentration-dependent reduction in viability activity, with significant results only for the highest concentration of diazepam (Fig. 1j). The extracts of St. John’s wort and valerian non-significantly lowered the viability of primary human lymphocytes under maximal, concentration conditions (Figs. 1a,c). The extracts of California poppy, lavender, and hops did not influence the viability of primary human lymphocytes (Figs. 1b,d,e). For hypericin and hyperforin present in St. John’s wort, as well as for valtrate present in valerian, a concentration-dependent reduction of viability was detected, with significant results for 3 µM of hypericin and hyperforin and 0.3 µM of valtrate (Figs. 1f,h). Protopine from California poppy, valerenic acid from valerian, and linalool from lavender did not show any changes in the viability of primary human lymphocytes (Figs. 1g,h,i).

### Effects of selected plant extracts and compounds on the induction of apoptosis in primary human lymphocytes

**Figure 2:**
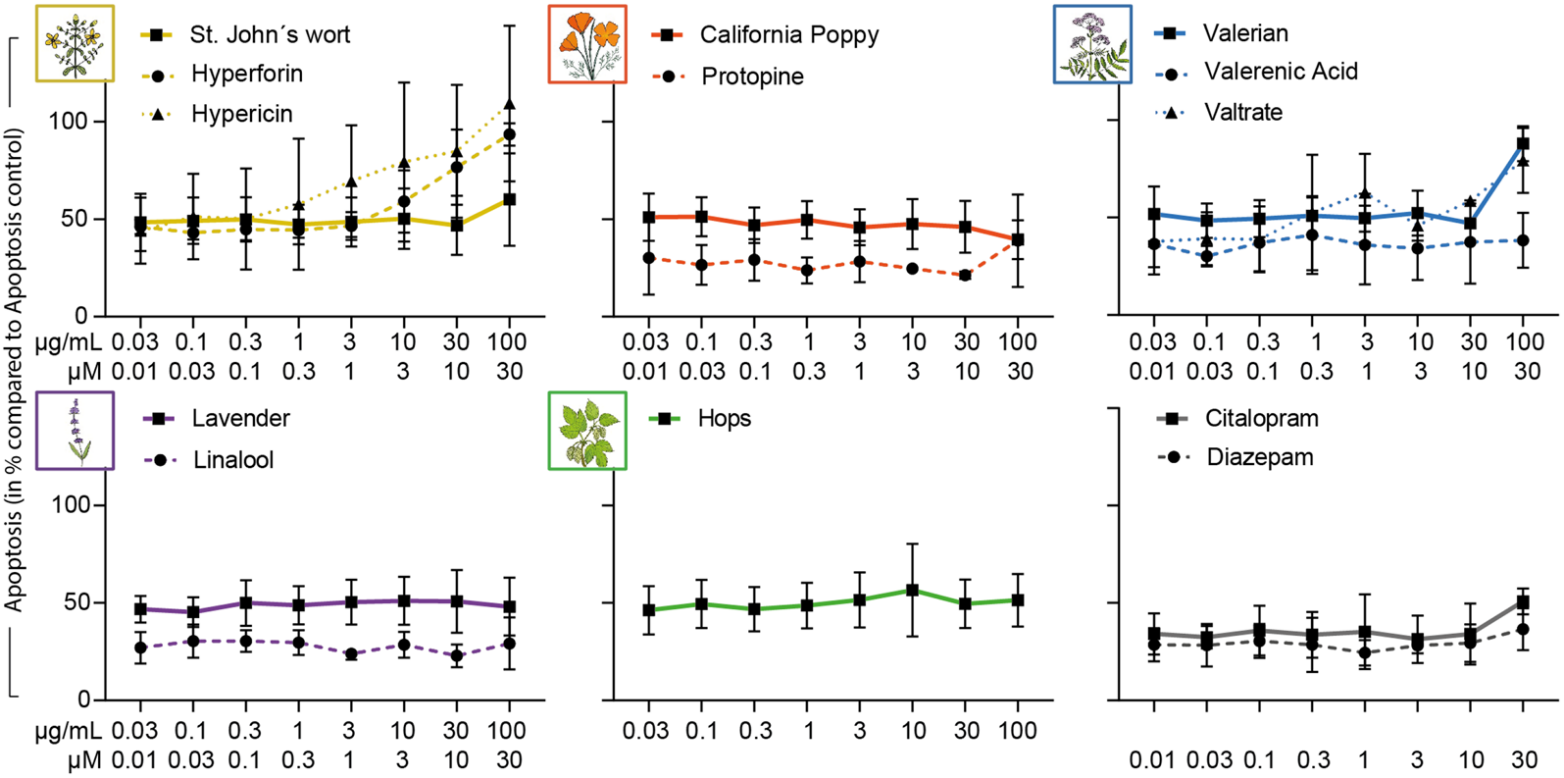
Effects of extracts and compounds on cell death (apoptosis) of stimulated primary human lymphocytes. Primary human lymphocytes were stimulated with anti-CD3 and anti-CD28 mAbs and incubated for 72 h with apoptosis control, extracts or compounds. Annexin V-FITC stainings were performed after incubation. The proportions apoptotic cells were determined by flow cytometry. Results were normalized to positive control for apoptosis CPT and depicted as mean ± standard deviation. *n* = 3.

In order to investigate the possible toxicity of the substances in a more differentiated way, their influence on the number of apoptotic cells was determined. For the controls diazepam and citalopram, as well as for the extracts of California poppy, lavender, and hops and their individual compounds protopine and linalool, no apoptosis-inducing effects could be detected in primary human lymphocytes. St. John’s wort and valerian increased the number of apoptotic cells only in the concentration of 100 µg/mL (Fig. 2). For hyperforin and hypericin, an elevated apoptosis rate could already be detected from a concentration of 0.3 µM and 3 µM, respectively (Fig. 2). Valerenic acid did not affect the number of apoptotic cells, whereas valtrate induced apoptosis at 10 µM (Fig. 2). However, none of the observed effects reached significance.

### Effects of selected plant extracts and compounds on the proliferation capacity of activated primary human lymphocytes

**Figure 3:**
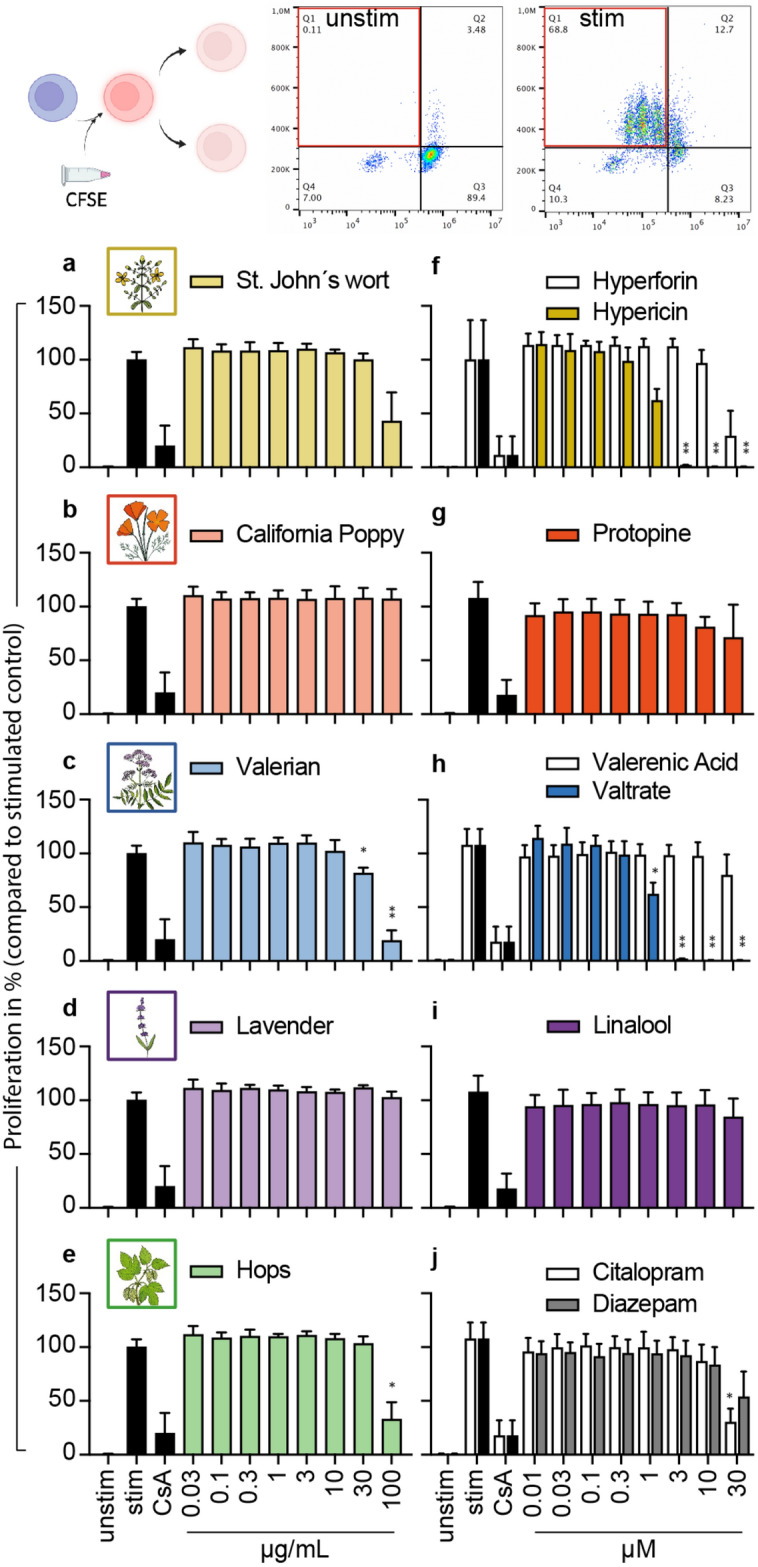
Effects of extracts and compounds on proliferation of stimulated primary human lymphocytes. Primary human lymphocytes were stained with CFSE and stimulated with anti-CD3 and anti-CD28 mAbs. Stimulated cells were incubated for 72 h in the presence of medium (unstim., stim.), cyclosporine A (CsA; 4.16 μM), extracts or compounds. Cell division was analyzed by flow cytometry. Results were normalized to positive control for proliferation inhibition CsA and depicted as mean ± standard deviation. *n* = 3; *p < 0.05; **p < 0.01.

Upon stimulus, primary human lymphocytes start to proliferate, which is essential for immune response. We therefore investigated the influence of selected substances on the cell division of primary human lymphocytes after stimulation, using CFSE, a dye that binds covalently to free amines and is distributed equally to daughter cells after cell division, in combination with flow cytometric analysis (Fig. 3). The controls citalopram and diazepam inhibited proliferation of activated primary human lymphocytes concentration-dependently, with a significant result for 30 µM citalopram (Fig. 3j). St. John’s wort suppressed the proliferation of primary human lymphocytes in a concentration-dependent manner, as did valerian, with significant results starting at 30 µg/mL (Figs. 3a,c). California poppy and lavender did not affect the cell division of primary human lymphocytes (Figs. 3b,d). For hops, proliferation inhibition was recorded only at the highest concentration of 100 µg/mL (Fig. 3e). Hyperforin and hypericin, the compounds from St. John’s wort, had concentration-dependent antiproliferative effects, with significant results for hypericin starting at a concentration of 3 µM (Fig. 3f). For valtrate from valerian, significant inhibition of the proliferation of primary human lymphocytes was observed, even at a concentration of 1 µM (Fig. 3h). The compounds protopine, valerenic acid, and linalool did not affect the cell division of primary human lymphocytes (Figs. 3g,h,i).

### Effects of selected plant extracts and compounds on the cell cycle progression of primary human lymphocytes

**Figure 4:**
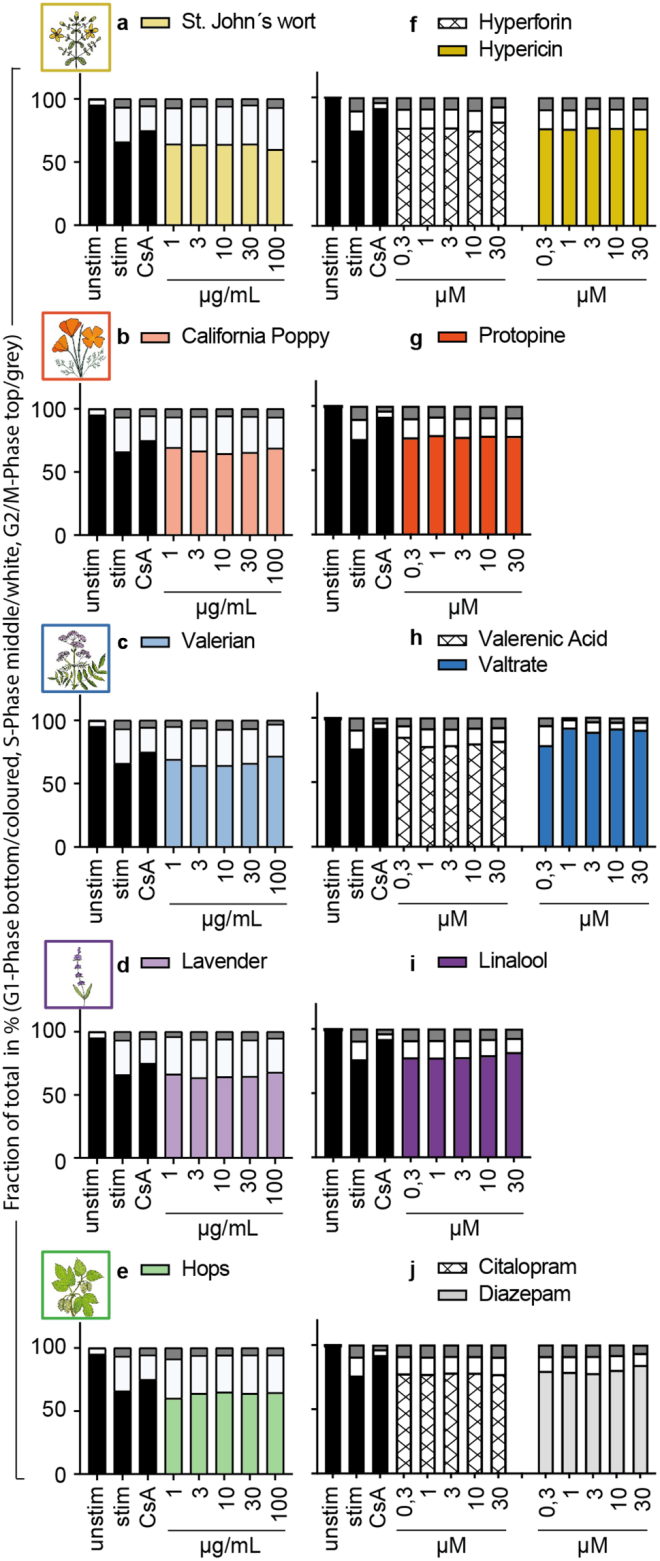
Effects of extracts and compounds on the cell cycle progression of primary human lymphocytes. The cell cycle was assessed after 72 h of incubation with medium (umstim., stim.), cyclosporine A (CsA; 4.16 μM), extracts or compounds via propidium iodide staining and measuring the fraction of cells in G1-Phase (single DNA set, depicted as the bottom/coloured part of the bar graph), S-Phase (duplication of DNA, the middle/white part) and G2/M-Phase (double set of DNA, the top/grey part). *n* = 3.

To investigate the mechanism of T cell proliferation inhibition, the cell cycle progression of primary human lymphocytes was analyzed for the plant extracts and compounds. The results showed no effect of the plant extracts (Figs. 4a,b,c,d,e), compounds (Figs. 4f,g,h,i), or controls (Fig. 4j) on the cell cycle of primary human lymphocytes. For valtrate (Fig. 4h), a trend was recognizable towards an arrest in the G1-Phase, comparable to the CsA inhibition control.

### Effects of selected plant extracts and compounds on the induction of genotoxicity in primary human lymphocytes

**Figure 5:**
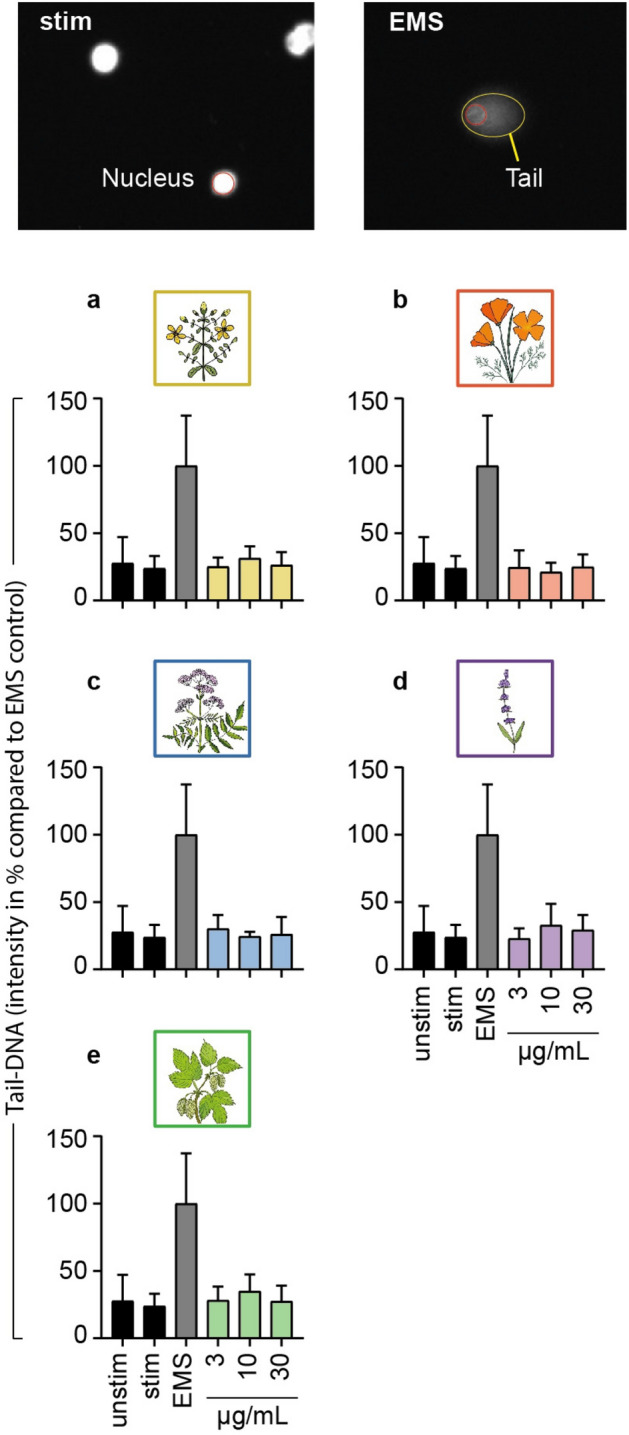
Effects of extracts on the induction of genotoxicity in primary human lymphocytes. Genotoxic potential of extracts was measured with single cell gel electrophoresis of primary human lymphocytes and analysis of nucleus to tail signal intensity ratio after exposure for 3 h. Results were calculated as tail-DNA intensity in % compared to the ethyl methanesulfonate (EMS, 3 mM) control. *n* = 3.

To evaluate the possible genotoxic damage of the plant extracts, comet assays using only non-cytotoxic concentrations were performed (Fig. 5). We performed the experiments only for the extracts since it cannot be assumed that the ingredients have a genotoxic effect if no genotoxicity was detected in the extracts. No DNA-damaging effect could be detected for any of the plant extracts (Figs. 5a,b,c,d,e).

### Effects of selected plant extracts and compounds on the functionality of primary human lymphocytes

**Figure 6:**
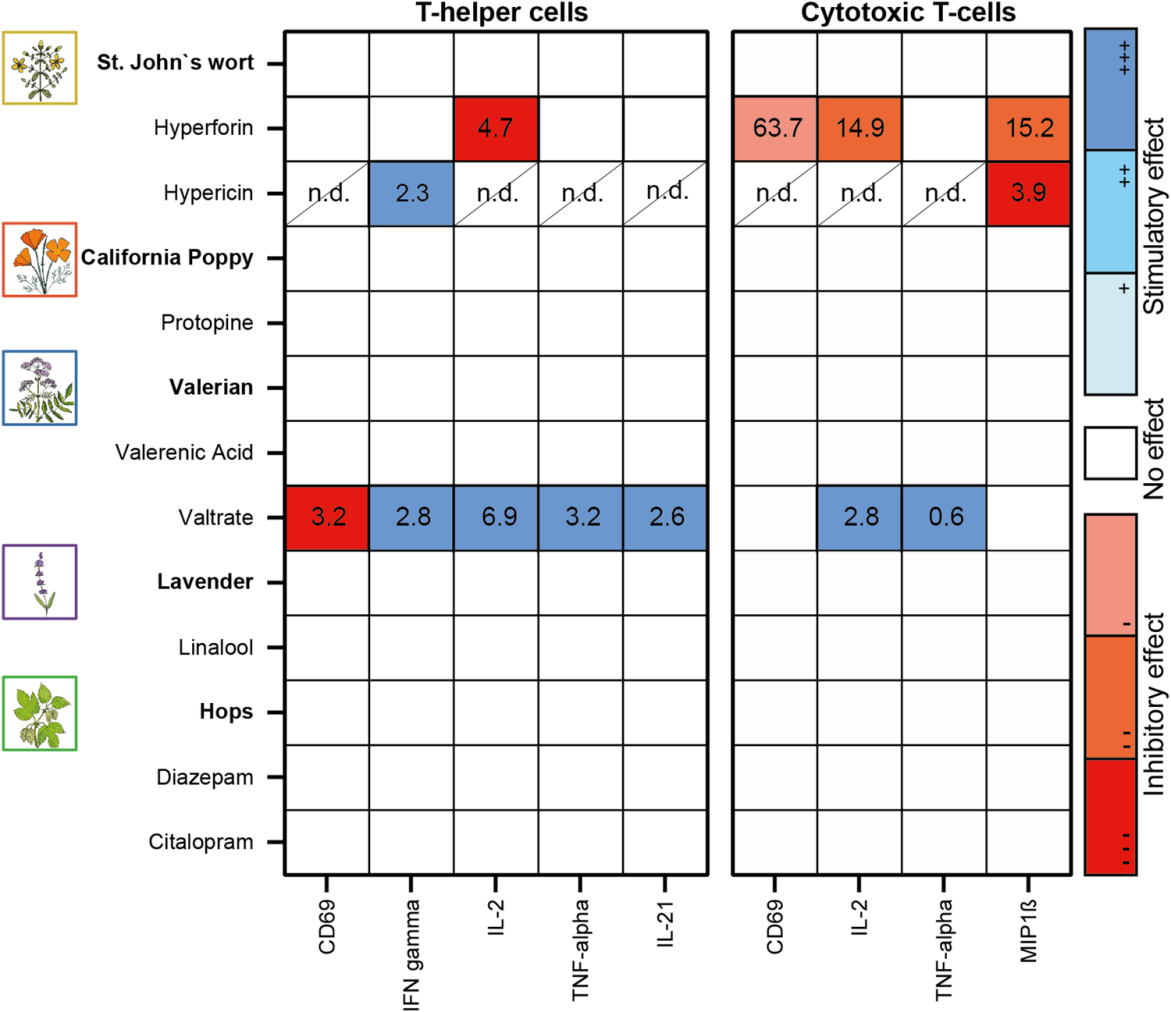
Effects of extracts and compounds on the activation state and cytokine production of primary human lymphocytes. After treatment with extracts and compounds for 44 h T-helper and cytotoxic T-cells were separately analyzed for their functional status using two flow cytometric staining panels. Activation markers (e.g., CD69) and cytokines were then compared to the stimulated control. To condense the available data the half maximal effective concentration (EC_50_ in µg/mL for extracts and µM for compounds) was calculated for all treatments and markers. In the heatmap only EC_50_ values are shown which originated from significant effects (red colour for inhibitory effects, blue colour for stimulatory effects). *n.d.*—non-detectable; *n* = 3.

The function of human T cells is characterized by the secretion of specific cytokines and surface markers that contribute to the promotion of the immune response. Here, a distinction must be made between the group of T helper cells and cytotoxic T cells. A multifluorescence panel assessed the expression of CD69, IFN-γ, IL-2, TNF-α, and IL-21 for T helper cells, and CD69, IL-2, TNF-α, and MIP1-β for cytotoxic T cells. The controls citalopram and diazepam did not affect the expression of the specific cytokines, nor did the extracts (Figure 6). The study of individual compounds showed differential effects of hypericin and hyperforin from St. John’s wort and valtrate from valerian. Hyperforin stimulated the expression of IFN-γ in contrast to the control but otherwise showed inhibitory effects on all measured parameters (Figure 6). Hypericin also stimulated the IFN-γ secretion and decreased the MIP1-β expression of cytotoxic T cells. The IL-21 expression of T helper cells was not affected by hypericin (Figure 6). The other parameters could not be analyzed for hypericin because the high autofluorescence of the compound interfered with the measurement, resulting in non-detectable values (n.d.). Valtrate from valerian gave completely opposite results. Although the activation of T helper cells was inhibited and that of cytotoxic T cells was not affected, valtrate had a stimulatory effect on all other parameters (Figure 6 and Supplementary Figure 1). For protopine, valerenic acid, and linalool, no influence on the investigated parameters was observed (Figure 6 and Supplementary Figure 1).

### Quantification of valerenic acid and valtrate in commercial preparations available in Switzerland

The previous results showed effects only for the compounds hyperforin and hypericin from St. John’s wort and valtrate from valerian. Therefore, the concentration of the compounds in commercial preparations from Switzerland was of interest to us in order to evaluate whether the observed effects could play a role in the use of the preparations for the treatment of NMDs during pregnancy. Because the data for St. John’s wort are already described in the literature^48^, only the valerian constituents valerenic acid and valtrate are quantified here (Table 1).

**Table 1:** Content of valerenic acid and valtrate in commercial preparations available in Switzerland. Data are reported as mg/100 mg of commercial preparation ± SD.

Formulation	Valerenic acid (n = 3)	Valtrate (n = 2)
Baldriparan	0.235 ± 0.009	< 0.001
Sedonium	0.098 ± 0.011	< 0.001
Zeller Schlaf Forte	0.071 ± 0.002	< 0.001
Arkopharma Valerian	0.168 ± 0.005	< 0.003

### Structural similarity of the investigated compounds with relevant control substances and immunosuppressants

In order to verify whether the effects of the investigated compounds might be based on mechanisms of action similar to those of the synthetic control substances and known immunoeffectors, in silico analyses were performed to assess their structural similarities. Two dimensional structural similarity was quantified using the Tanimoto score based on extended FP2 fingerprints.

For hyperforin and valerenic acid, the in silico analyses showed a moderate structural similarity to the corticosteroids prednisone and prednisolone (Table 2). For hyperforin, a structural similarity to corticosteroid budesonide was also detected (Table 2). For valtrate, structural analyses revealed some structural similarities to the mTOR inhibitors sirolimus and everolimus, as well as the calcineurin inhibitor tacrolimus.

**Table 2:** Table of 2D similarity scores (Tanimoto) calculated using OpenBabel program.

Reference substances	Californidine	Hyperforin	Hypericin	Linalool	Protopine	Valerenic acid	Valtrate
Ethyl-Methanesulfonate	0.03	0.04	0.06	0.09	0.02	0.05	0.02
Camptothecin	0.15	0.08	0.14	0.04	0.19	0.06	0.14
Cyclosporin-A	0.11	0.28	0.10	0.26	0.13	0.24	0.17
Staurosporin	0.18	0.10	0.12	0.07	0.20	0.08	0.22
Diazepam	0.13	0.04	0.16	0.02	0.13	0.04	0.09
Citalopram	0.22	0.06	0.17	0.09	0.19	0.06	0.11
Prednisone	0.07	**0.61**	0.12	0.33	0.11	**0.62**	0.21
Budensonide	0.10	*0.43*	0.13	0.22	0.13	0.39	0.39
Prednisolone	0.07	**0.64**	0.12	0.38	0.11	**0.62**	0.23
Cyclosporin	0.11	0.28	0.10	0.26	0.13	0.24	0.17
Tacrolimus	0.14	0.25	0.10	0.14	0.16	0.20	*0.42*
Voclosporin	0.11	0.27	0.10	0.24	0.13	0.23	0.19
Sirolimus	0.13	0.25	0.10	0.14	0.15	0.20	*0.41*
Everolimus	0.13	0.24	0.09	0.14	0.15	0.19	*0.43*
Azathioprine	0.12	0.07	0.08	0.05	0.14	0.06	0.11
Leflumide	0.13	0.05	0.13	0.03	0.17	0.05	0.07
Mycophenolate	0.22	0.21	0.38	0.15	0.28	0.16	0.23
Anakinra	0.13	0.08	0.10	0.04	0.14	0.09	0.15
Maraviroc	0.25	0.15	0.16	0.08	0.25	0.11	0.16
Plerixafor	0.28	0.07	0.15	0.07	0.23	0.06	0.11
Cyclophosphamid	0.08	0.07	0.06	0.09	0.08	0.09	0.07
Hydroxychloroquin	0.16	0.07	0.11	0.07	0.14	0.07	0.08
Methotrexate	0.19	0.10	0.13	0.06	0.22	0.09	0.16

Significant values above 40 are given in bold and Values below 40 are given in bold-italic.

## Discussion

Occurring in approximately 15% of pregnant women, NMDs can have serious consequences for both mother and child^1,3–6^. Therapy with standard medications, such as antidepressants (SSRIs) and benzodiazepines (GABAA receptor modulators), is challenging because they can cross the placenta and harm the unborn child^7,9,10^. Commercially available herbal preparations are a possible treatment alternative. The present study is part of a project^63^ evaluating the safety of taking commercially available herbal preparations for the treatment of NMDs. The influence on the viability and function of primary human lymphocytes of St. John’s wort, valerian, hops, lavender, and California poppy, and their compounds hyperforin and hypericin, protopine, valerenic acid, and valtrate, as well as linalool, was investigated. This is significant because a balance between fetal tolerance and the protective function of the immune system is important during pregnancy.

None of the total extracts at the physiological concentration studied showed a significant effect on the viability or function of primary human lymphocytes. In addition, no induction of apoptosis or genotoxicity was found for any of the extracts. A significant inhibitory effect on the proliferation of primary human lymphocytes could only be detected for St. John’s wort and valerian at a concentration of 30 µg/mL. For St. John’s wort, anti-inflammatory effects are already described in the literature and are thus in line with the data of this study^40–47^. For valerian, little is known with respect to immunomodulation. Only an influence on the innate immune system and ROS production is known^52^. Thus, the first evidence of valerian’s potential to modulate the adaptive immune system is presented here. The immunological effects described for lavender were not supported in this study^59^.

The compounds protopine, valerenic acid, and linalool also showed no significant effects on viability, proliferation, induction of apoptosis or genotoxicity, or the function of primary human lymphocytes. The compounds hyperforin, hypericin, and valtrate inhibited viability, induced apoptosis, and inhibited cell division with effects from a concentration of 3 µM. Differential effects were seen with respect to function. Hyperforin and hypericin stimulated IFN-γ production but otherwise showed inhibitory effects on cytokine production by T helper cells and cytotoxic T cells. In contrast, valtrate decreased T helper cell activation marker expression but had no effect on cytotoxic T cell activation markers. However, stimulatory effects were found for all other markers examined.

The present study shows that no T cell modulatory effects were detected when California poppy, lavender, and hops are used in vitro. In the experiments performed, neither the total extracts nor the protopine and linalool compounds affected the viability, proliferation, and function of primary human lymphocytes. For the use of St. John’s wort and valerian for the treatment of NMDs during pregnancy, the concentration of the compounds hyperforin and hypericin, as well as valtrate, in the products available on the market must be carefully evaluated, as in some cases stronger effects occurred here.

Because published data on bioavailability and metabolization are limited to date, a bioavailability of 100% and no metabolization was initially assumed. To calculate the maximum concentration of plant extracts in the body fluids of pregnant women, we assumed about 31.1 L of body fluid in 30-year-old women, calculated for an average body weight of 63 kg^64^ and a body height of 165 cm^65^ (the body fluids was calculated using the Watson formula). The maximum recommended dose for St. John’s wort, valerian, hops, lavender, and California poppy preparations is between 80 and 1200 mg/day (highest value is related to the maximal dose of Somnofor^66^).

Data on hypericin and hyperforin concentrations in St. John’s wort products available in the Swiss market are known from the literature. According to the data, the highest hypericin concentration was 0.21 mg/100 mg (Sandoz Hypericum) and that of hyperforin 1.62 mg/100 mg (Hyperiplant)^48^. Assuming a maximum daily dose of 1200 mg and a body fluid volume of 31.1 L, the maximum concentrations would be 0.16 µM and 1.17 µM for hypericin and hyperforin, respectively. However, it is improbable that calculated concentrations in the body fluid will actually be reached, supported by a small number of studies investigating the bioavailability of the relevant compounds.

Thus, in a pharmacokinetics study in healthy volunteers, a hypericin plasma concentration of 16.6 ng/L (0.033 µM) is determined after a single dose of 1500 µg hypericin^67^. For hyperforin, a plasma concentration of 437.3 ng/mL (0.815 µM) is reported after a dose of 1200 mg of St. John's wort extract (corresponding to 59.2 mg hyperforin)^68^. Since the significant effects of hypericin and hyperforin occurred only at concentrations above 3 µM, no effects on the viability and function of primary human lymphocytes are expected from the intake of St. John’s wort preparations. For valerenic acid and valtrate, the concentrations in preparations available on the Swiss market were determined in this study. The highest concentration of valerenic acid was 0.235 mg/ 100 mg in the product Baldriparan. A study on 31 commercial preparations of valerian, mostly from the Australian market, found valerenic acid concentrations ranging from 0.001 to 0.632 mg/100 mg^69^. The Baldriparan concentration of valerenic acid corresponds to a maximum concentration of 0.38 µM in the body fluids (maximum daily dose of 1200 mg and a body fluid volume of 31.1 L). Considering pharmacokinetics data in healthy volunteers from a published study, it was calculated that after a single dose of 300 mg valerian (corresponding to 1569 µg valerenic acid), a plasma concentration of 3.3 ng/ml (0.014 µM) can be expected^70^. Valtrate was virtually absent in the preparations investigated here and mostly not detectable in 31 differents preparations of valerian^69^. In the product Arkopharma Valerian, a concentration of < 0.003 mg/100 mg was measured. This corresponds to a concentration of < 0.003 µM when a maximum of 1200 mg is taken (body fluid volume of 31.1 L). Thus, both valerenic acid and valtrate were so low in concentration when the valerian preparations were taken that the effects observed in this study on the viability and function of primary human lymphocytes were not relevant.

With regard to further studies, the mechanism of action of the relevant ingredients is also of great interest in assessing global effects and interactions with other drugs. The structure of the substances is decisive for their function and mode of action^71,72^. In order to get an impression of the structure of the relevant ingredients, and thus of their potential mode of action, *in-silico* analyses, comparing the structure of herbal substances with the structure of relevant control substances and known immunosuppressants, were performed in this study. Hyperforin and valerenic acid showed structural similarities to the glucocorticoids prednisone and prednisolone, as well as budesonides, in the case of hyperforin. Glucocorticoids are steroid hormones that can cross the cell membrane and then bind to and activate their intracellular glucocorticoid receptors (GR). The activated receptor is transported into the nucleus, where it binds to DNA to regulate many lymphocyte genes (e.g., nuclear factor ‘kappa-light-chain-enhancer’ of activated B-cells (NF-κB), activator protein 1 (AP-1), or signal transducers and activators of transcription (STAT)), thereby suppressing lymphocyte function^73,74^. In addition, glucocorticoids can induce apoptosis and decrease the expression of adhesion molecules for lymphocyte migration^75^. A moderate structural similarity to glucocorticoids could indicate a comparable mechanism of action. The interaction of the non-steroidal natural product antcin A from *Antrodia camphorata* with GR is known, and furthermore, the synthesis of non-steroidal selective glucocorticoid receptor modulators is still a focus of research^76,77^. Consequently, an interaction of the nonsteroidal substances hyperforin and valerenic acid on GR would be possible and could be the subject of future investigations. Valtrate showed some structural similarity to the compounds sirolimus and everolimus as well as tacrolimus. All compounds directly affect T cell signaling, either via blockage of the mammalian target of rapamycin (mTor) activation (sirolimus and everolimus^78,79^), or via inhibition of the phosphatase calcineurin (tacrolimus^78^). Consequently, it is quite possible that valtrate also directly affects T-cell signaling. However interestingly, in contrast to the mentioned compounds valtrate shows an induction of selected cytokine production. An effect that needs additional attention as it would suggest a more diverse mode of action than immunosuppression alone. This should also be clarified in subsequent studies.

This study investigates the influence of commercially available herbal preparations for the treatment of NMDs on defined subsets of human immune cells in vitro. This is relevant for physicians and patients because the fragile immunological balance during pregnancy is very important to ensure fetal tolerance and, at the same time, an adequate defense against infections. The substances St. John’s wort, valerian, hops, lavender, and California poppy and their compounds hyperforin and hypericin, protopine, linalool, valerenic acid, and valtrate most likely do not pose a threat to immune cells in vitro. However, additional studies confirming these findings in preclinical and clinical studies as well as investigating potential metabolites will be necessary for a conclusive safety assessment.

## Methods

### Ethics approval statement

All subjects gave written informed consent for blood collection. The blood samples were obtained in an anonymized and coded form from the central blood donation of the University Hospital in Basel. No ID number of the samples is visible, so that any assignment is impossible. The work therefore does not fall within the scope of the Swiss Human Research Act. Thus, no ethics vote by the Ethics Committee Central and Northwestern Switzerland is required for the methods used to work with the blood samples.

### Isolation and cell culture of human peripheral lymphocytes

Preparation and cultivation of human peripheral lymphocytes was performed as indicated in^80^.To obtain Peripheral Blood Mononuclear Cell (PBMCs), blood donations of healthy donors were submitted to density gradient centrifugation with Lymphoprep^TM^ (Stemcell Technologies) sugar gradient media (density: 1.077 g/cm^3^, 20 min, 500 g, 20 °C; Progen). After centrifugation, the white blood cells were extracted and washed twice with phosphate-buffered saline (PBS, GE Healthcare). Finally, the PBMCs were transferred into culture medium: RPMI 1640 medium supplemented with 10% fetal calf serum (FCS, GE Healthcare Life Sciences), 2 mM L-glutamine, 100 U/mL penicillin, and 100 U/mL streptomycin (all from Sigma-Aldrich). The cells were cultured in an incubator at 37 °C, 5 % CO_2_, and 95 % air atmosphere.

### Treatment with extracts or compounds

In preparation of the experimental treatment, the extracts and pure compounds hyperforin, and linalool (Sigma-Aldrich), hypericin (Carbosynth), protopine and valerenic acid (Extrasynthese), and valtrate (Toronto Research Chemicals) were dissolved in sterile DMSO (Sigma-Aldrich), aliquoted and stored for later use at –80 °C. In the experimental setups PBMCs were treated with a variety of concentrations for extracts (0.03–100 µg/mL) and pure compounds (0.01–30 µM) in culture medium. Hyperforin and hypericin experimentation was performed under minimal light to avoid possible deterioration of the substances. As synthetic controls citalopram and diazepam were used in similar concentrations as the pure compounds. All cells were stimulated with 100 ng/mL CD3 and CD28 monoclonal antibodies (mAbs) (eBioscience), except for the unstimulated control cells. The herbal extracts used were previously prepared and characterized^66^. All experiments were repeated at least 3 times with different donor material (n=3).

### Viability assay

A spectrometric assay was used to assess the viability of cells. Cells were seeded at a concentration of 2*10^5^ cells/mL. Test substances were added immediately after stimulation. CsA (5 µg/mL; Sandimmun®) was used as inhibition control. The cells were incubated for 72 h under normal culture conditions, washed and treated with the tetrazolium salt WST-1 (Roche) for 2 h. Absorption of the metabolized formazan dye was carried out at 450 nm using a microplate reader (Tecan Infinite M200).

### Cell death assay (Apoptosis)

Determination of apoptosis of T cells was performed as previously reported^80^. PBMCs were treated with extracts, compounds and CPT (300 µM; apoptosis control; Tocris Bioscience). After 72 h incubation time the cells were stained with AnnexinV-FITC (eBioscience) as recommended by the manufacturer`s instructions. The fraction of apoptotic cells was determined via flow cytometric readout using a CytoflexS (Beckman Coulter) and FlowJo Software.

### Proliferation assay

The proliferation of T lymphocytes was determined using carboxyfluorescein diacetate succinimidyl ester (CFSE) staining, as described earlier^81,82^. After isolation, PBMCs were washed with PBS and set to a concentration of 5*10^6^ cells/mL in preparation of staining (10 min at 37 °C) with CFSE (5 μM; Sigma-Aldrich). Excess CFSE was washed away with culture medium after which the cells were counted again and set to normal experimental conditions (2*10^5^ cells/mL with 100 µL/well in a 96-well plate). After 72 h incubation time, measurement was performed using a CytoflexS (Beckman Coulter) and FlowJo Software.

### Cell cycle assay

Cells (2*10^6^ cells/mL) were treated for 72 h at 37 °C/5% CO_2_. Subsequently, the cells were washed with cold (4°C) PBS, and slowly resuspended with 3 mL ice cold 70% ethanol. The PBMCs were then kept at −20°C overnight for fixation. Afterwards, cells were washed with PBS and resuspended in 200 µL staining solution (1µL Propidium iodide (1mg/mL; Sigma-Aldrich) in 199 µL PBS). After staining of the cells for 30 min at room temperature (RT) in the dark, measurement was carried out using a CytoflexS (Beckman Coulter).

### Comet assay (DNA damage)

The genotoxic potential of the extracts was investigated using a single cell DNA gel electrophoresis. To avoid possible DNA-repair mechanisms, cells were treated for 3 h only. As DNA-damage control 3 mM ethyl methanesulfonate (EMS) (positive control; Sigma-Aldrich) was used. In preparation, microscopic slides had been coated with 1% normal-melting agarose in PBS (NMA, SERVA Electrophoresis GmbH). During the incubation time a slightly boiling 0.7% NMA solution (200 µL) was applied to the precoated slides and cooled on ice cold aluminium sheets. For the final layer 0.7% low melting agarose (LMA SERVA Electrophoresis GmbH) was heated to 100 °C and then kept at 38 °C. for later use. After incubation, the cells were centrifuged and resuspended in 30 µL culture medium. These concentrated cell aliquots were then mixed with 90 µL LMA, immediately transferred onto the prepared slides and again cooled on the aluminium sheets. After the LMA hardened, the cells were lysed and afterwards the electrophoresis was run at 25 V/300mA for 20 min. The slides were washed with ddH_2_O, PBS and fixed with pure ethanol. The fixed samples were stored in the fridge until staining with ethidium bromide solution (5 µg/mL; Carl Roth GmbH) and imaging with a microscope. Analysis of the images was performed with CometScore software (version 2.0.038 for Windows; TriTek Corp., USA).

### Immunological multifluorescence panel

PBMCs were set to 5*10^6^ cells/mL, stimulated and treated with extracts and pure compounds for 40 h. Cells were restimulated with phorbol-12-myristat-13-acetate (PMA, 50 ng/mL; Sigma-Aldrich) and ionomycin (1 µg/mL; Sigma-Aldrich), except for unstimulated controls. Additionally, the golgi apparatus was blocked with GolgiPlug^TM^ (1 µL/mL; BD Biosciences) and GolgiStop^TM^ (0.65 µL/mL; BD Biosciences) to inhibit export of cytokines and cells were incubated for another 4 h. Surface staining mix was prepared for 2 separate panels (Table 3):

**Table 3:** Surface antibodies used for multifluorescence staining.

Panel 1		Panel 2	
CD3-APC AlexaFluor750	Beckman-Coulter	CD3-APC AlexaFluor750	Beckman-Coulter
CD4-AlexaFluor700	Beckman-Coulter	CD8-AlexaFluor700	Beckman-Coulter
CD69-PC7	Beckman-Coulter	CD69-PC7	Beckman-Coulter

Cells were stained for 30 min at RT in the dark and resuspended in 100 µL Cytofix/Cytoperm solution for 15 min at 4 °C. The intracellular staining mix was prepared (Table 4):

**Table 4:** Intracellular antibodies used for multifluorescence staining.

Panel 1		Panel 2	
IFN-γ FITC	Beckman-Coulter	IFN-γ FITC	Beckman-Coulter
IL-2 APC	BD	TNF-α PE	Beckman-Coulter
TNF-α PE	Beckman-Coulter	MIP1-β BV421	Beckman-Coulter
IL-21 BV421	BD		BD

Cells were stained for 30 min at 4 °C. Afterwards, the cells were fixed with 2% paraformaldehyde (PFA; Electron Microscopy Sciences) for 10 min at 4 °C.

Fluorescence intensity of the cells was measured with a CytoflexS flow cytometer (Beckman Coulter). Analysis of the data was performed using FlowJo software and the half maximal effective values (EC_50_) were calculated with GraphPad software.

### Quantification of valerenic acid and valtrate by HPLC

Valerenic acid was purchased from Phytolab and valtrate from Toronto Research Chemicals Inc. Valerian preparations included different galenic forms. Baldriparan and Sedonium consisted of pills and were purchased from PharmaSGP GmbH and Vifor SA, respectively. Zeller Schlaf Forte were film-coated tablets and were purchased from Zeller AG. Arkopharma Valerian consisted of capsules containing powdered valerian root and was purchased from Arkopharma. Arkopharma Valerian capsules were extracted by pressurized liquid extraction with 70% EtOH in a Dionex ASE 200 Accelerated Solvent Extractor. Three cycles of extraction of 5 min each at a temperature of 70 °C and a pressure of 120 bar were applied. The sample was evaporated under reduced pressure and lyophilized to give a powder.

Pills and tablets (after removal of coating) were grinded. The powders were weighed separately, dissolved in DMSO at a concentration of 20 mg/mL, sonicated for 15 min, and centrifuged at 3500 rpm for 20 min. Supernatant was collected and transferred into an HPLC vial for analysis. The samples were analyzed on an LC‑MS system consisting of an 8030 triple quadrupole mass spectrometer connected to an HPLC system composed of a DGU-20A degasser, an LC-20AD binary high-pressure mixing pump, a SIL-20 ACHT autosampler, a CTO-20AC column oven, and an SPD-M20A diode array detector (all Shimadzu). Analyses were performed at 25°C on a SunFire C18 column (3.5 μm; 150×3 mm i.d., Waters). For valerenic acid, the mobile phase consisted of water (A) and acetonitrile (B), both containing 0.1% formic acid. For valtrate, the mobile phase consisted of water containing 10 mM ammonium formate (A) and acetonitrile (B), both supplemented with 0.05% formic acid. All samples were analyzed with a 5–100% B gradient in 30 min at a flow rate of 0.4 mL/min. Calibration samples were prepared in DMSO. For valerenic acid, a calibration curve was made with concentrations ranging from 1 to 100 µg/mL. Detection was at UV 223 nm. Calibration samples of valtrate were ranging from 0.1 to 10 µg/mL. Detection was performed with ESI-MS in positive ion mode, using the extracted ion trace at m/z 445.5, corresponding to the sodium ion adduct of valtrate.

### Structural similarity of the investigated compounds with relevant control substances and immunosuppressants

Structural information (SDF format) of seven studied natural compounds as well as selected reference compounds were obtained from the PubChem (https://pubchem.ncbi.nlm.nih.gov/) repository using trivial names as text search queries. OpenBabel software version 3.0 (http://openbabel.org, ^83^) was used to convert the SDF files to the SMILES format and to calculate the similarity scores of all natural compounds against all reference compounds. The similarity was quantified using the Tanimoto score based on FP2 extended fingerprints (indexing linear fragments up to 7 atoms).

### Statistical data analysis

Statistical data analysis was performed using PRISM (version 9.3.1 for PC; GraphPad Software). Normality was tested using Shapiro-Wilk test and was given in most cases. Since the Welch ANOVA is generally very robust to violations of a normal distribution, all sample concentration were tested in a multiple comparison with the Welch ANOVA and Dunnet´s T3 test against the stimulated control. Statistical significance was considered for *p < 0.05, **p < 0.01, ***p < 0.001, ****p < 0.0001. This strategy was chosen for the statistical analysis due to the number of repetitions per experiment, the expected dose dependent results and different Standard Deviations (SDs) per concentration.

## Supplementary Information

Supplementary Information.

## Data Availability

The datasets generated for this study are available on request to the corresponding author.

## Abbreviations

NMDs: Nonpsychotic mental diseases
SSRIs: Selective serotonin reuptake inhibitors
GABAA: γ-Aminobutyric acid type A
IL: Interleukin
Tregs: Regulatory T cells
FoxP3: Forkhead box P3
AMPK: AMP-activated protein kinase
ROS: Reactive oxygen species
GR: Glucocorticoid receptor
NF-κB: Nuclear factor 'kappa-light-chain-enhancer' of activated B-cells
AP-1: Activator protein 1
STAT: Signal Transducers and Activators of Transcription
mTor: Mammalian target of rapamycin
CFSE: Carboxyfluorescein succinimidyl ester
EMS: Ethyl methanesulfonate
PBMCs: Peripheral blood mononuclear cells
mAbs: Monoclonal antibodies
CsA: Cyclosporin A
CPT: Camptothecin
PMA: Phorbol-12-myristat-13-acetate
