# Supplementary material for: Immunological evaluation of herbal extracts commonly used for treatment of mental diseases during pregnancy
Source: Sci Rep. 2023 Jun 14;13:9630. doi: 10.1038/s41598-023-35952-5 (PMC10267184; doi:10.1038/s41598-023-35952-5)
Supplement: Supplementary file 1 — Supplementary Information. [file 41598_2023_35952_MOESM1_ESM.docx]

**Supplementary Information**

**Immunological evaluation of herbal extracts commonly used for treatment of mental diseases during pregnancy**

Moritz Winker^1^, Antoine Chauveau^2^, Martin Smieško^3^, Olivier Potterat^2^, Alexander Areesanan^1^, Amy Zimmermann-Klemd^1*^, Carsten Gründemann^1*^

^1^ Translational Complementary Medicine, Department of Pharmaceutical Sciences, University of Basel, Basel, Switzerland

^2^ Division of Pharmaceutical Biology, Department of Pharmaceutical Sciences, University of Basel, Basel, Switzerland

^3^ Computational Pharmacy, Department of Pharmaceutical Sciences, University of Basel, Basel, Switzerland

**Supplementary Figure 1: Activation state and cytokine production of primary human lymphocytes.** After treatment with extracts and compounds for 44 h T-helper and cytotoxic T-cells were separately analyzed for their functional status using flow cytometric staining panels. Activation markers (e.g., CD69) and cytokines were then compared to the stimulated control. n.d. = non-detectable; n=3.
